# Stable Gastric Pentadecapeptide BPC 157 Heals Established Vesicovaginal Fistula and Counteracts Stone Formation in Rats

**DOI:** 10.3390/biomedicines9091206

**Published:** 2021-09-13

**Authors:** Domagoj Rasic, Anita Zenko Sever, Fran Rasic, Sanja Strbe, Zarko Rasic, Antonija Djuzel, Bozidar Duplancic, Alenka Boban Blagaic, Anita Skrtic, Sven Seiwerth, Predrag Sikiric, Marko Sever

**Affiliations:** 1 Department of Urology, School of Medicine, University of Zagreb, Salata 3b, 10000 Zagreb, Croatia; domagoj.rasic89@gmail.com; 2 Department of Pathology, School of Medicine, University of Zagreb, Salata 9, 10000 Zagreb, Croatia; anita.zenko@gmail.com (A.Z.S.); sven.seiwerth@mef.hr (S.S.); 3 Department of Pharmacology, School of Medicine, University of Zagreb, POB 916, Salata 11, 10000 Zagreb, Croatia; fran.rasic@icloud.com (F.R.); strbes@gmail.com (S.S.); antonija.djuzel@gmail.com (A.D.); abblagaic@mef.hr (A.B.B.); 4 Department of Surgery, School of Medicine, University of Zagreb, Salata 3b, 10000 Zagreb, Croatia; zarko.rasic@zg.t-com.hr (Z.R.); dr.sever.marko@gmail.com (M.S.); 5 Department of Anaesthesia, School of Medicine, 21000 Split, Croatia; bozidarduplancic@gmail.com

**Keywords:** stable gastric pentadecapeptide BPC 157, vesicovaginal fistula, stone formation, rats

## Abstract

With the stable gastric pentadecapeptide BPC 157 therapy known to heal various both external and internal rat fistulas, we attempt to approach vesicovaginal fistula, continuous urine leaking through vagina, bladder stones, and a possible therapy solution among rats with well-formed 2 week-fistulas (vaginal/vesical 4 mm large defects) started with delayed therapy. Subsequent control fistula course (the subsequent 1, 2, 4, and 6 weeks) since beginning revealed the failed healing, fistula leaking, adhesions, urinary leaking through vagina, failed epithelization, collagenization, granulation tissue and neovascularization, increased inflammation, and necrosis. Thereby, the later intervals revealed the persistent inability to sustain even minimal volume, vesical, and vaginal defects and stone formation at the end of the experiment (fistula-time day 56). BPC 157 therapy (10 µg/kg, 10 ng/kg, intraperitoneally once time daily or perorally in drinking water until sacrifice) was initiated with a considerable delay (at 2 weeks after fistula formation). Already within 1 week therapy, all BPC 157 regimens stopped urinary leaking through vagina, reversed the otherwise resistant poor healing course to the increased epithelization, collagenization, granulation tissue and neovascularization, decreased inflammation, and decreased necrosis. Thereby, at later intervals, all BPC 157 rats exhibited a five times larger volume that can be sustained before leaking as in healthy, vesical, and vaginal defects completely closed and no stone formation. Thus, macro/microscopic and functional recovery, and counteracted stone formation. Concluding, BPC 157 therapy’s beneficial effects resulted in healing and no stone formation, with µg- and ng-regimens, either given daily perorally in drinking water or intraperitoneally.

## 1. Introduction

With the stable gastric pentadecapeptide BPC 157 therapy (for review, see, i.e., [1,2,3,4]), known to heal various both external [5,6,7,8] and internal [9,10] rat fistulas, we attempt to approach vesicovaginal fistula, continuous urine leaking through vagina, and bladder stones [11,12,13,14], as well as a possible therapy solution. Patients with a vesicovaginal fistula, the most common form of genitourinary fistula [11,14], typically present with continuous vaginal urine drainage [14], and smell of urine [15], and the degree of urinary incontinence is typically proportional to the size of the fistula tract [16], and likely presentation of the bladder stone that may further erode fistula as well. The proposed mechanisms for vesicovaginal fistula include vaginal ischemia from obstetric compression, obstetric-related tearing or shearing of the vagina and surrounding tissue, and finally iatrogenic trauma related to gynecologic surgery [14]. Thereby, the rat vesicovaginal fistula that would spontaneously not heal, made by anastomosing the bladder and vaginal defects, may fairly mimic the continuous fistulas course in the patients. The failed spontaneous healing capability would allow the delayed therapy of the well-established fistulas. Moreover, providing the rat’s vaginal size [10], and defect/vagina size relation [10], this may even correlate the high size of the fistula as well, and thereby the special therapy effect.

Of note, as an especial point, the proposed BPC 157 effectiveness in the resistant vesicovaginal fistulas goes with its wound healing potential, recently reviewed (i.e., skin, muscle, tendon, ligament, bone; ulcers in the entire gastrointestinal tract; corneal ulcer; lethal dose (LD1) is not achieved) [4,17], and molecular pathways implicated in the healing [18,19,20,21,22,23,24,25,26,27], in the fistulas curing in particular [3]. Thereby, there is the assumption that the healing of the various wounds is equally essential for both gastrointestinal fistulas and urogenital fistulas healing [3,4]. Documented consistent healing of the various gastrointestinal fistulas, external (esophagocutaneous, gastrocutaneous, duodenocutaneous, and colocutaneous) [5,6,7,8] and internal (colovesical, rectovaginal) [9,10], was ascribed to its original healing capacity as a peptide native and stable in human gastric juice, and its possible role as novel mediator of Robert’s cytoprotection maintaining gastrointestinal mucosal integrity [3], as it is effective in the whole gastrointestinal tract and used in ulcerative colitis clinical trials [1,2,3,4]. Taking fistulas as a pathological connection, and BPC 157 to reestablish original organ function [3], this particular rescue is verified with the beneficial effects in the rats with the various gastrointestinal anastomoses (i.e., esophagogastric [28], jejunoileal [29,30], colo-colonic [31], ileoileal [32], esophagojejunal [33], esophagoduodenal [34], and gastrojejunal [35]) as well as vessel [36] and nerve [37] anastomoses.

Thereby, the BPC 157 healing effect on fistulas in rats may be relevant also for the special injurious conditions that may arise in the resistant prolonged vesicovaginal fistulas. This may be further supported by BPC 157 therapeutic potentials, given intraperitoneally, or perorally, in drinking water, for the colovesical or rectovaginal fistulas [9,10], and leak point pressure recovery in rat stress urinary incontinence after transabdominal urethrolysis and prolonged vaginal dilatation [38]. Likewise, there is the evidence that BPC 157 given intraperitoneally, or perorally, in drinking water, may counteract cyclophosphamide-induced hemorrhagic cystitis, grossly, microscopically, and functionally [39].

Thus, in the previous BPC 157-fistulas studies, the therapy was initiated soon after fistulas formation [5,6,7,8,9,10], as a further advent in fistulas therapy; whereas in this study with the vesicovaginal fistulas, unable to spontaneously heal for a long period of the 7 weeks, started after 2 weeks following fistulas formation. Since that time, the rats with well-formed vesicovaginal fistulas started with the BPC 157 therapy given intraperitoneally, or perorally, in drinking water.

## 2. Materials and Methods

### 2.1. Animals

This study was conducted with 12 weeks old, 200 g body weight, female albino Wistar rats, randomly assigned at 6 rats/group/interval. Rats were bred in-house at the Pharmacology Animal Facility, School of Medicine, Zagreb, Croatia. The animal facility was registered by the Directorate of Veterinary (reg. no. HR-POK-007). Laboratory rats were acclimated for five days and randomly assigned to their respective treatment groups. Laboratory animals were housed in polycarbonate (PC) cages under conventional laboratory conditions at 20–24 °C, relative humidity of 40–70% and noise level 60 dB. Each cage was identified with dates, number of study, group, dose, number, and sex of each animal. Fluorescent lighting provided illumination 12 h per day. Standard good laboratory practice (GLP) diet and fresh water was provided ad libitum. Animal care was in compliance with standard operating procedures (SOPs) of the pharmacology animal facility, and the European Convention for the Protection of Vertebrate Animals used for Experimental and other Scientific Purposes (ETS 123).

This study was approved by the local Ethic Committee. Ethical principles of the study complied with the European Directive 010/63/E, the Law on Amendments to the Animal Protection Act (Official Gazette 37/13), the Animal Protection Act (Official Gazette 135/06), the Ordinance on the protection of animals used for scientific purposes (Official Gazette 55/13), Federation of European Laboratory Animal Science Associations (FELASA) recommendations and the recommendations of the Ethics Committee of the School of Medicine, University of Zagreb. The experiments were assessed by observers blinded as to the treatment.

### 2.2. Drugs

Medication was administered as described previously [6,7,8,9,10], without use of a carrier or peptidase inhibitor, for stable gastric pentadecapeptide BPC 157, a partial sequence of the human gastric juice protein BPC, which was freely soluble in water at pH 7.0 and in saline. BPC 157 (GEPPPGKPADDAGLV, molecular weight 1419; Diagen, Ljubljana, Slovenia) was prepared as a peptide with 99% high-performance liquid chromatography (HPLC) purity, with 1-des-Gly peptide being the main impurity. The dose and application regimens were as described previously [5,6,7,8,9,10].

### 2.3. Procedure

Animals were anesthetized with Ketamine 20 mg/kg and Apaurin 6 mg/kg given intraperitoneally. After standard surgical preparation of the operating field (shaving of the abdominal skin, washing and disinfection of the operating field with povidone-iodide), skin and muscle incision in the lower median line was made, 3–4 cm in length, and the abdominal cavity was approached. The bladder, uterus with adnexa, and vagina were shown. A longitudinal incision was made in the posterior wall of the bladder and the anterior wall of the vagina in a length of 4 mm. Vesicovaginal fistula was formed by single suture technique (Vycril 4-0). The length of the fistula was controlled by a movable scale. The abdominal wall was closed in layers with individual sutures (Vycril 3-0 for muscles, silk 3-0 for skin).

### 2.4. Experimental Protocol after Surgery

Therapy regimens starting at the day 14 after fistula creation (therapy day 0), included BPC 157, dissolved in saline, 10 µg/kg or 10 ng/kg given once daily intraperitoneally, last application at 24 h before sacrifice, or in drinking water (0.16 µg/mL/rat/day, 0.16 ng/mL/rat/day) until the sacrifice. Controls received simultaneously an equal volume of saline (5 mL/kg) intraperitoneally or drinking water (12 mL/rat/day). Sacrifice was after 7, 14, 21, 28, and 42 therapy days, or at the day 21, 28, 35, 42, and 56 after fistula creation, respectively.

### 2.5. Vesicovaginal Fistula Assessment Protocol

Vesicovaginal fistula assessment protocol was as in the previous fistula studies [5,6,7,8,9,10], the rectovaginal fistulas [10], in particular, as follows.

#### 2.5.1. Fistula Leakage

To assess fistula leakage [5,6,7,8,9,10] and the closure of the fistula, we assessed the volume (mL) that was sustained before the initiation of the leakage through the fistula. The volume of saline was infused through a syringe-perfusion pump system (Argus 600; Argus Medical A6, Heimberg, Switzerland) at the rate of 1 mL/10 s. The infusion was stopped at the point when the leakage through the external aperture of the fistula started. If there was no leaking till the end of the fifth minute, the fistula was considered to be functionally closed.

#### 2.5.2. Urinary Leaking through Vagina

All rats were observed for urinary leaking through vagina, upon gentle pressure to rat abdomen.

#### 2.5.3. Adhesion

Adhesion presentation was scored 0–7: 0—no increments; 1—thin adhesions cover less than half of the fistula; 2—more prominent adhesions covering more than half of the fistula; 3—solid adhesions that cover the entire fistula; 4—adhesions include the uterus and bladder; 5—adjacent intestinal meanders covered by adhesions; 6—adhesions in the lower half of the abdominal cavity; 7—all organs are covered by appendages.

#### 2.5.4. Microlithiasis or Stone Formation in Bladder

Assessment verified presence or absence at the time of the sacrifice.

#### 2.5.5. Vesical Defect, Vaginal Defect, Fistula Assessment

Briefly, a precise caliper was used to verify the final size of the defect and the largest diameter of the vesical and vaginal defect was assessed (mm), photographed and further verified using the program ISSA (VAMSTEC Software Company, Zagreb, Croatia) as described before [5,6,7,8,9,10].

The tissue was processed for further microscopic analysis [5,6,7,8,9,10]. Immediately after sacrifice, bladder and vaginal tissue samples were taken in area of the formed fistula, fixed with styrofoam pins with minimal tension, and fixed with formalin according to an already known protocol [5,6,7,8,9,10]. Samples were cut and stained with hematoxylin and eosin. SFORM and ISSA software programs manufactured by VAMSTEC-Software Company (Zagreb, Croatia) were used for morphometric analysis. Five fields were selected for analysis under high magnification by randomization. The samples were analyzed by experienced observers who were unfamiliar with the origin of the material, with a micrometer mounted on a lens at a magnification of 200 times, and under a 10 × 100 magnification optical microscope. The data collected consisted of an average of 8 separate measurements per animal. Histopathological findings were scored as described before [10]: epithelialization (1—none; 2—partial; 3—complete, immature; 4—complete, mature); collagenization (1—none; 2—partial; 3—complete, irregular; 4—complete, regular); inflammation (1—none; 2—mild; 3—moderate; 4—severe,); necrosis (1—focal; 2—abundant) and granulation tissue (1—no; 2—immature; 3—slightly mature; 4—moderately mature; 5—fully mature).

### 2.6. Statistical Analyses

Statistical analysis was performed by a non-parametric Kruskal–Wallis ANOVA and subsequent Mann–Whitney U test to compare groups. Fisher’s exact probability test for urinary leakage through vagina, and presence of the microlithiasis or stone in bladder rate assessment was used. Values of *p* ˂ 0.05 were considered statistically significant.

## 3. Results

Rats underwent vesicovaginal fistulas presented a complex course. Fistula leaking, adhesions, urinary leaking through vagina, epithelization, collagenization, inflammation, granulation tissue, neovascularization, and necrosis presented undisturbed poor course, and thereby persistent vesical and vaginal defects and stone formation. Initiated with a considerable delay (at 2 weeks after fistula formation), BPC 157 therapy reversed the otherwise inevitably poor healing course to the consistent closure of both defects in all BPC 157 rats, microscopic and functional recovery, and no stone formation. Thus, beneficial effect of BPC 157 therapy resulted in the healing, µg- and ng-regimens, either given daily perorally in drinking water or intraperitoneally.

### 3.1. Fistula Leakage

**Figure 1:**
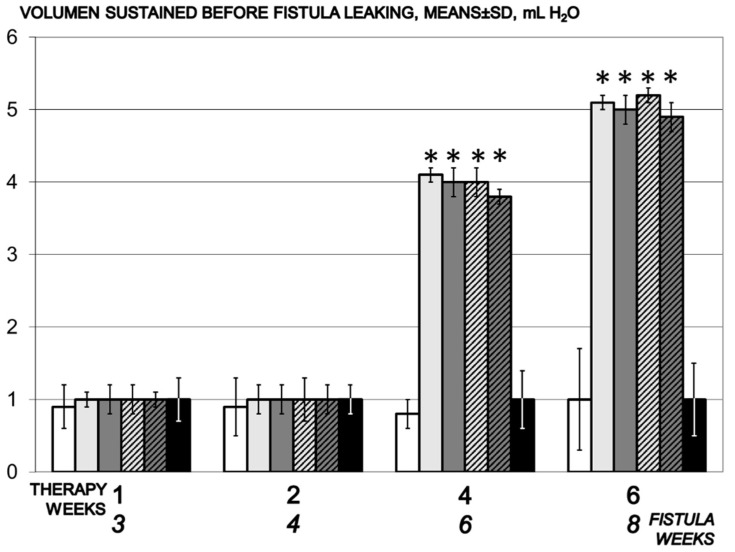
Volume that could be sustained before fistula leakage, mL H2O, means ± SD, time line. Therapy regimens starting at the day 14 after fistula creation (therapy day 0), included BPC 157 given once daily intraperitoneally, last application at 24 h before sacrifice, 10 µg/kg (light gray bars) or 10 ng/kg (dark gray bars) or given perorally, in drinking water 10 µg/kg/day (dashed light gray bars) or 10 ng/kg (dashed dark gray bars) until the sacrifice. Controls received simultaneously an equal volume of saline (5 mL/kg) intraperitoneally (white bars) or drinking water (12 mL/rat/day) (black bars). * *p* < 0.05, relative to control.

The effect was seen at the fourth week of the therapy. Thereafter, the maximal instilled volume reached the values of healthy rats, and was five times larger than in the small volume sustained before fistula leakage in the controls (Figure 1).

### 3.2. Urinary Leaking through Vagina

**Figure 2:**
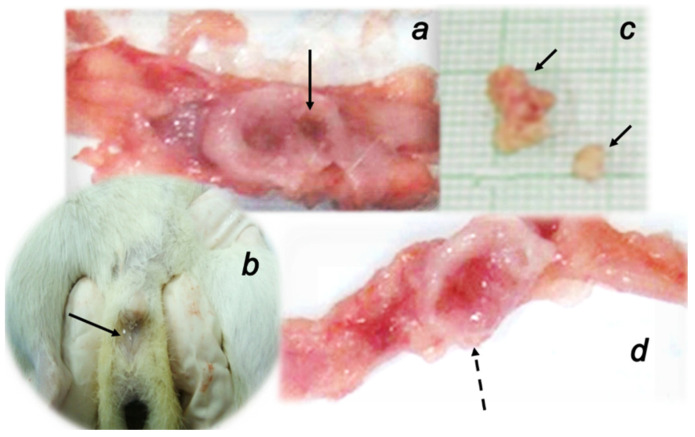
Gross presentation of rats with vesicovaginal fistulas. Bladder presentation and lesion (defect) (**a**), urinary leaking through the vagina (**b**), vesical stones (**c**) (full arrows) always noted in all control rats. These disturbances were absent in rats treated with BPC 157 regimens presenting with the preserved bladder presentation (**d**) (dashed arrow).

Urinary leaking through the vagina was constant in the controls until the end of the experiment. In contrast, urinary leaking through the vagina was stopped in all BPC 157 treated fistulous rats already at the first week of the therapy, and never reappeared (Fisher exact probability test *p* ˂ 0.05, at least vs. control) (Figure 2).

### 3.3. Microlithiasis or Stone Formation in Bladder

All controls presented with the stone in the bladder when duration of the vesicovaginal fistulas is prolonged (i.e., vesicovaginal fistula day 42 or 56). In contrast, BPC 157 treated fistulous rats had no bladder stone (Fisher exact probability test *p* ˂ 0.05, at least vs. control) (Figure 2).

### 3.4. Adhesions

**Figure 3:**
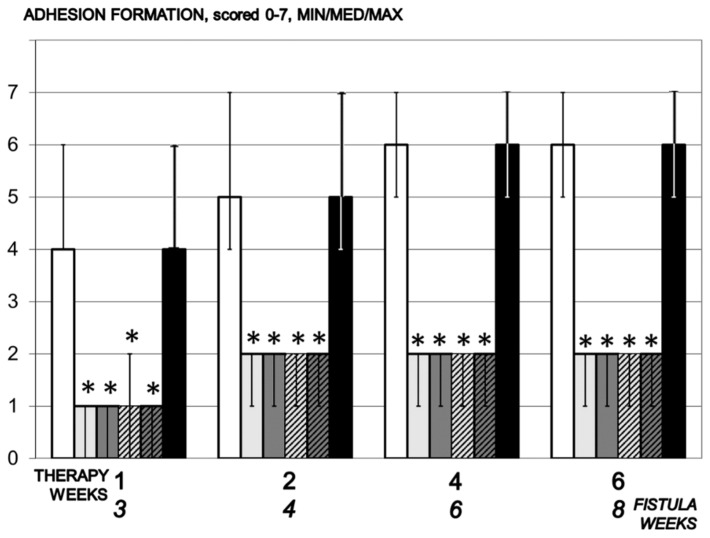
Adhesion formation, scored 0–7, Min/Med/Max, time line. Therapy regimens starting at the day 14 after fistula creation (therapy day 0), included BPC 157 given once daily intraperitoneally, last application at 24 h before sacrifice, 10 µg/kg (light gray bars) or 10 ng/kg (dark gray bars) or given perorally, in drinking water 10 µg/kg/day (dashed light gray bars) or 10 ng/kg (dashed dark gray bars) until the sacrifice. Controls received simultaneously an equal volume of saline (5 mL/kg) intraperitoneally (white bars) or drinking water (12 mL/rat/day) (black bars). * *p* < 0.05, relative to control.

Regularly, considerable adhesions occurred after vesicovaginal fistula formation, as seen in the controls without therapy. Contrarily, since the very early therapy time, the adhesion formation was markedly attenuated in the fistulous rats treated with BPC 157 regimens (Figure 3).

### 3.5. Fistula

#### 3.5.1. Vesical Defect, Vaginal Defect, Fistula Assessment

**Figure 4:**
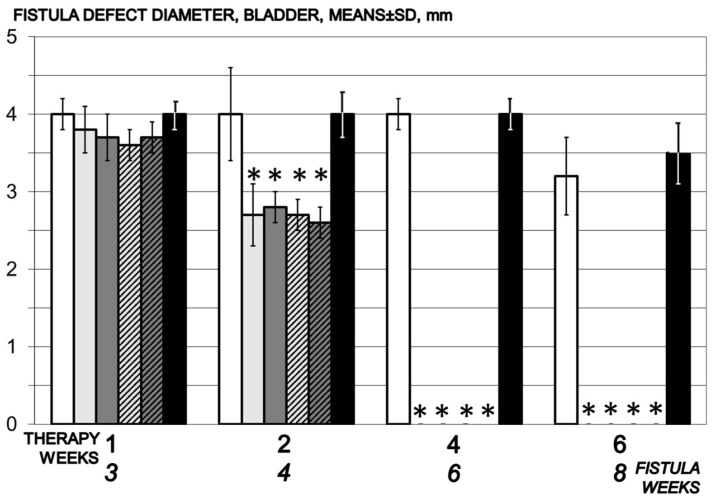
Bladder defect diameter, mm, means ± SD, time line. Therapy regimens starting at the day 14 after fistula creation (therapy day 0), included BPC 157 given once daily intraperitoneally, last application at 24 h before sacrifice, 10 µg/kg (light gray bars) or 10 ng/kg (dark gray bars) or given perorally, in drinking water 10 µg/kg/day (dashed light gray bars) or 10 ng/kg (dashed dark gray bars) until the sacrifice. Controls received simultaneously an equal volume of saline (5 mL/kg) intraperitoneally (white bars) or drinking water (12 mL/rat/day) (black bars). * *p* < 0.05, relative to control.

**Figure 5:**
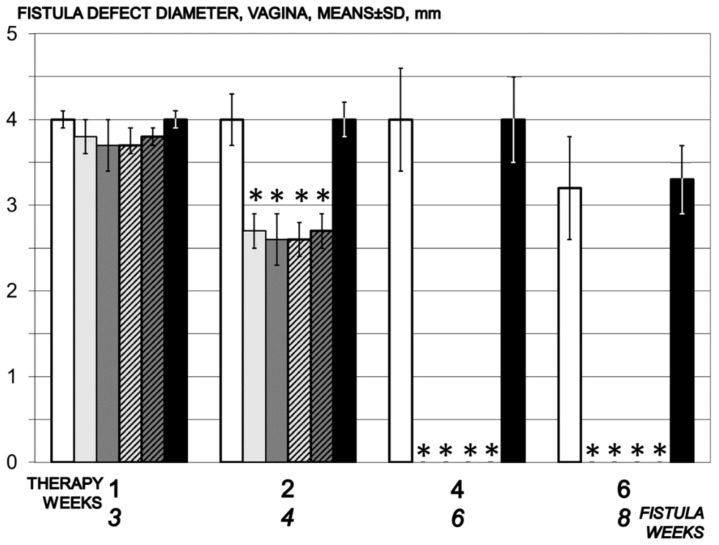
Vaginal defect diameter, mm, means ± SD, time line. Therapy regimens starting at the day 14 after fistula creation (therapy day 0), included BPC 157 given once daily intraperitoneally, last application at 24 h before sacrifice, 10 µg/kg (light gray bars) or 10 ng/kg (dark gray bars) or given perorally, in drinking water 10 µg/kg/day (dashed light gray bars) or 10 ng/kg (dashed dark gray bars) until the sacrifice. Controls received simultaneously an equal volume of saline (5 mL/kg) intraperitoneally (white bars) or drinking water (12 mL/rat/day) (black bars). * *p* < 0.05, relative to control.

Two weeks of therapy BPC 157 for rats presented decrease of the vaginal and vesical defects, and complete closure after four weeks of the therapy (Figure 4 and Figure 5). 

Interestingly, in addition to less adhesion formation, antecedents are the effect on the epithelization, collagenization, inflammation, granulation tissue, neovascularization, and necrosis.

#### 3.5.2. Epithelization

**Figure 6:**
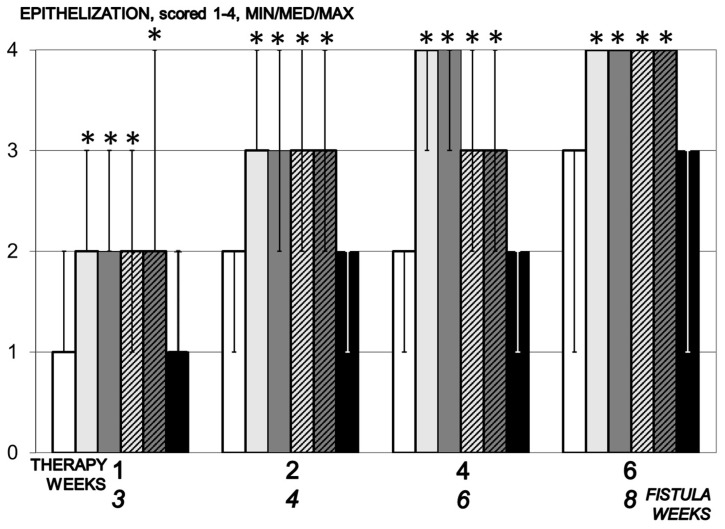
Epithelization, scored 1–4, Min/Med/Max, time line. Therapy regimens starting at the day 14 after fistula creation (therapy day 0), included BPC 157 given once daily intraperitoneally, last application at 24 h before sacrifice, 10 µg/kg (light gray bars) or 10 ng/kg (dark gray bars) or given perorally, in drinking water 10 µg/kg/day (dashed light gray bars) or 10 ng/kg (dashed dark gray bars) until the sacrifice. Controls received simultaneously an equal volume of saline (5 mL/kg) intraperitoneally (white bars) or drinking water (12 mL/rat/day) (black bars). * *p* < 0.05, relative to control.

Illustratively, 7 days therapy epithelialization is complete within next 7 days, and complete tissue maturation within three weeks’ therapy. In control, epithelialization begins on day 14 of the experiment and is incomplete throughout the experiment (Figure 6).

#### 3.5.3. Collagenization

**Figure 7:**
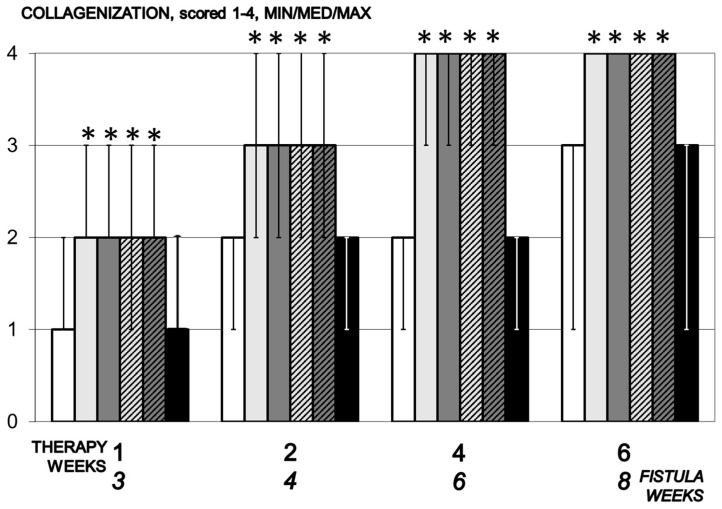
Collagenization, scored 1–4, Min/Med/Max, time line. Therapy regimens starting at the day 14 after fistula creation (therapy day 0), included BPC 157 given once daily intraperitoneally, last application at 24 h before sacrifice, 10 µg/kg (light gray bars) or 10 ng/kg (dark gray bars) or given perorally, in drinking water 10 µg/kg/day (dashed light gray bars) or 10 ng/kg (dashed dark gray bars) until the sacrifice. Controls received simultaneously an equal volume of saline (5 mL/kg) intraperitoneally (white bars) or drinking water (12 mL/rat/day) (black bars). * *p* < 0.05, relative to control.

Collagen formation in animals treated with BPC 157 begins on day 7 of the experiment and fully formed collagen was within four and five weeks of therapy. In the control group, collagen begins to form between days 14 and 28 (Figure 7).

#### 3.5.4. Inflammation

**Figure 8:**
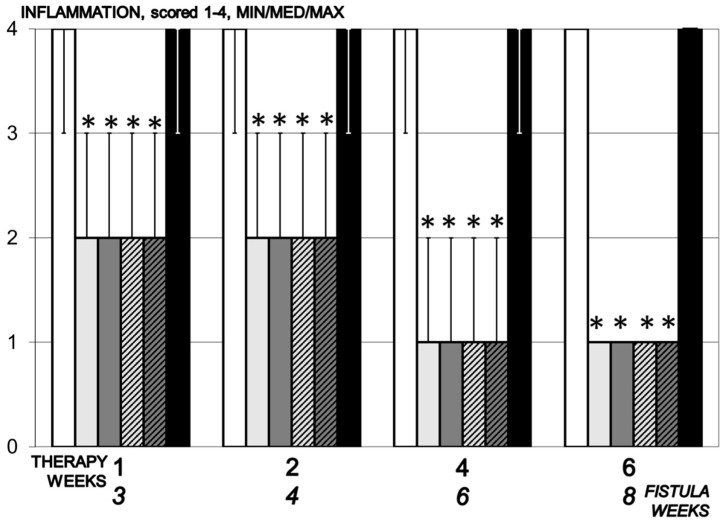
Inflammation, scored 1–4, min/med/max, time line. Therapy regimens starting at the day 14 after fistula creation (therapy day 0), included BPC 157 given once daily intraperitoneally, last application at 24 h before sacrifice, 10 µg/kg (light gray bars) or 10 ng/kg (dark gray bars) or given perorally, in drinking water 10 µg/kg/day (dashed light gray bars) or 10 ng/kg (dashed dark gray bars) until the sacrifice. Controls received simultaneously an equal volume of saline (5 mL/kg) intraperitoneally (white bars) or drinking water (12 mL/rat/day) (black bars). * *p* < 0.05, relative to control.

In the control, significant inflammation was observed within the first two weeks, moderate after four weeks and mild inflammation after six weeks, while in animals treated with BPC 157 moderate to mild inflammation was found after day 7 with regression of inflammation by the end of the experiment (Figure 8).

#### 3.5.5. Neovascularization

**Figure 9:**
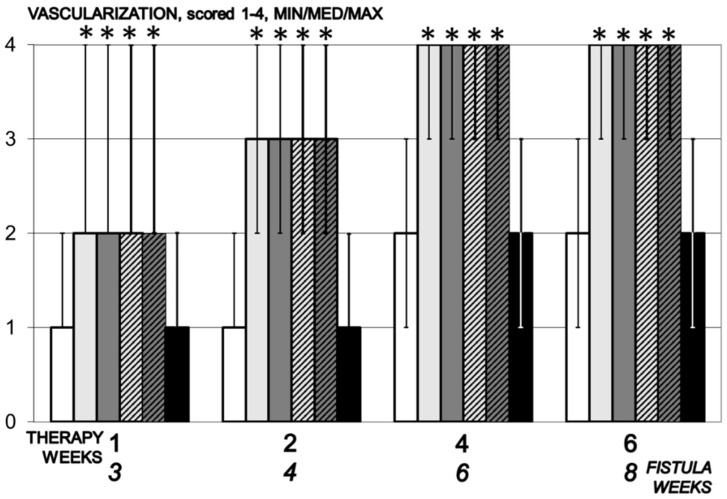
Vascularization, scored 1–4, min/med/max, time line. Therapy regimens starting at the day 14 after fistula creation (therapy day 0), included BPC 157 given once daily intraperitoneally, last application at 24 h before sacrifice, 10 µg/kg (light gray bars) or 10 ng/kg (dark gray bars) or given perorally, in drinking water 10 µg/kg/day (dashed light gray bars) or 10 ng/kg (dashed dark gray bars) until the sacrifice. Controls received simultaneously an equal volume of saline (5 mL/kg) intraperitoneally (white bars) or drinking water (12 mL/rat/day) (black bars). * *p* < 0.05, relative to control.

In control animals, the neovascularization process was slowed down, while in the group receiving BPC 157 it occurred from day 7, resulting in the formation of new blood vessels within four weeks of starting therapy (Figure 9).

#### 3.5.6. Necrosis

**Figure 10:**
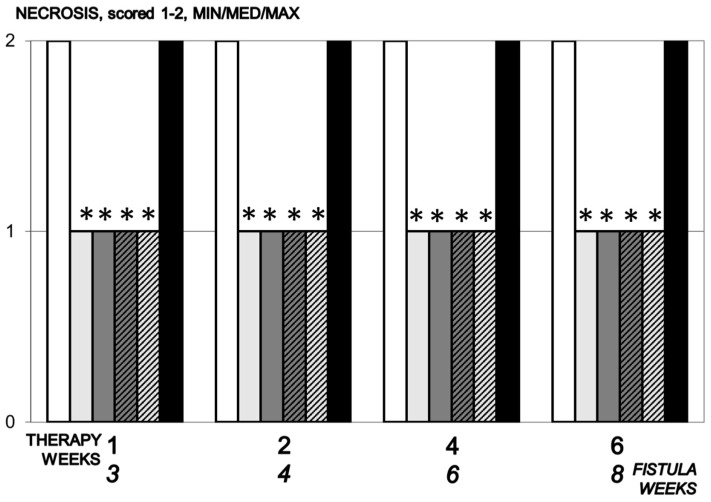
Necrosis, scored 1–2, Min/Med/Max, time line. Therapy regimens starting at the day 14 after fistula creation (therapy day 0), included BPC 157 given once daily intraperitoneally, last application at 24 h before sacrifice, 10 µg/kg (light gray bars) or 10 ng/kg (dark gray bars) or given perorally, in drinking water 10 µg/kg/day (dashed light gray bars) or 10 ng/kg (dashed dark gray bars) until the sacrifice. Controls received simultaneously an equal volume of saline (5 mL/kg) intraperitoneally (white bars) or drinking water (12 mL/rat/day) (black bars). * *p* < 0.05, relative to control.

A great number of focal necrosis was observed in control animals over two weeks, while no focal necrosis was observed or was very mild in BPC 157 treated animals (Figure 10).

#### 3.5.7. Granulation

**Figure 11:**
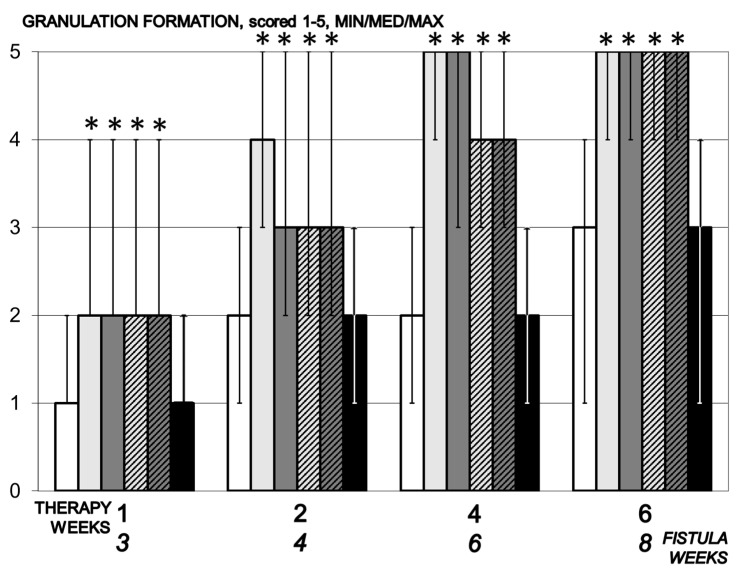
Granulation formation, scored 1–5, min/med/max, time line. Therapy regimens starting at the day 14 after fistula creation (therapy day 0), included BPC 157 given once daily intraperitoneally, last application at 24 h before sacrifice, 10 µg/kg (light gray bars) or 10 ng/kg (dark gray bars) or given perorally, in drinking water 10 µg/kg/day (dashed light gray bars) or 10 ng/kg (dashed dark gray bars) until the sacrifice. Controls received simultaneously an equal volume of saline (5 mL/kg) intraperitoneally (white bars) or drinking water (12 mL/rat/day) (black bars). * *p* < 0.05, relative to control.

Granulation tissue occurs in control animals only on day 14, while in groups receiving BPC 157 it is observed from day 7 and mature granulation tissue is present over 14 days (Figure 11).

### 3.6. Microscopy Presentation

**Figure 12:**
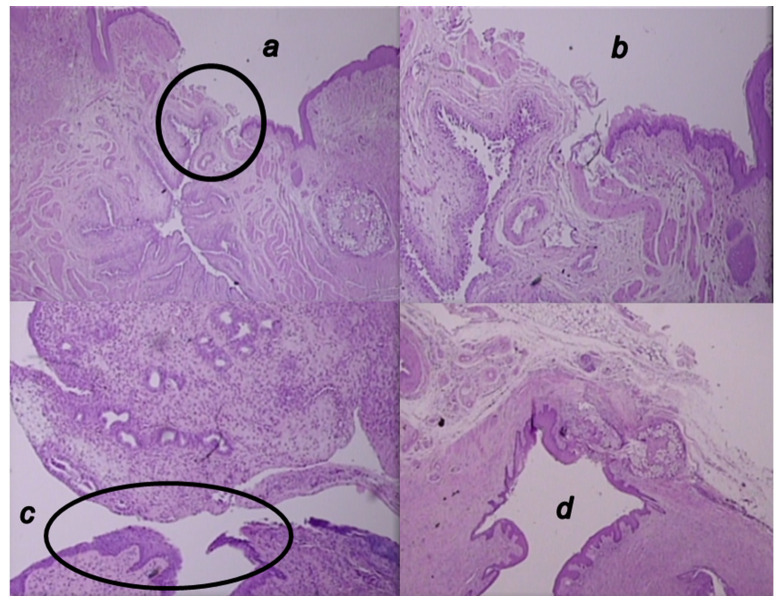
Microscopic presentation of 4 weeks-vesicovaginal fistula, after the two-week therapy of fistulous rats in the BPC 157 treated rats (**a**,**b**) and control rats (**c**,**d**). (**a**,**b**). Visible significant complete epithelialization (circle), collagenization, neovascularization, and formation of granulation tissue in the BPC 157-rats (HE; ×20 (**a**), ×100 (**b**)). (**c**,**d**). Visible open vesicovaginal fistula, tissue defect at the site of the fistula (circle, (**c**)) with pronounced inflammation (mononuclear cells and neutrophils), and with significant stromal edema in the controls (**d**) (HE; ×100 (**c**), ×20 (**d**)).

**Figure 13:**
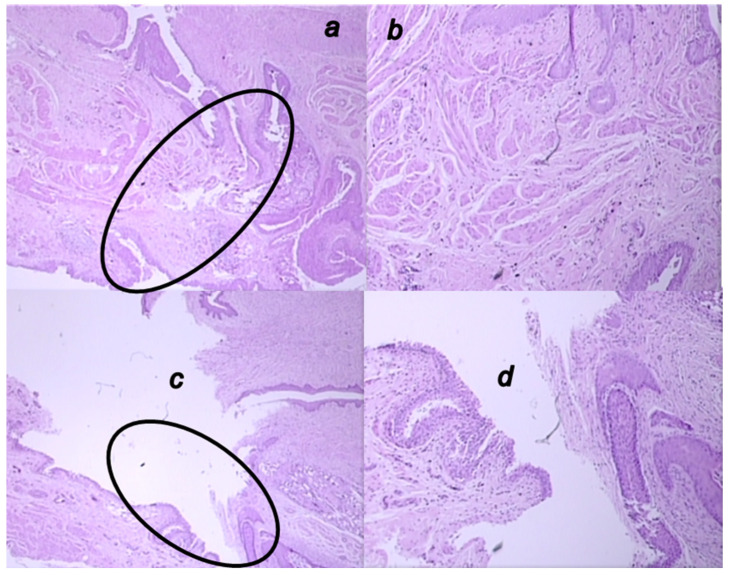
Microscopic presentation of 6 weeks-vesicovaginal fistula, after the 4 weeks-therapy of fistulous rats in the BPC 157 treated rats (**a**,**b**) and control rats (**c**,**d**). (**a**,**b**) Significant neovascularization with formation of new blood vessels (circle) and mature granulation tissue is visible in the BPC 157-rats (HE; ×20 (**a**), ×100 (**b**)). (**c**,**d**). Visible open vesicovaginal fistula in control (circle) with moderate inflammation, mature epithelialization, and fully formed subepithelial collagen tissue in the controls (HE; ×20 (**c**), ×100 (**d**)).

**Figure 14:**
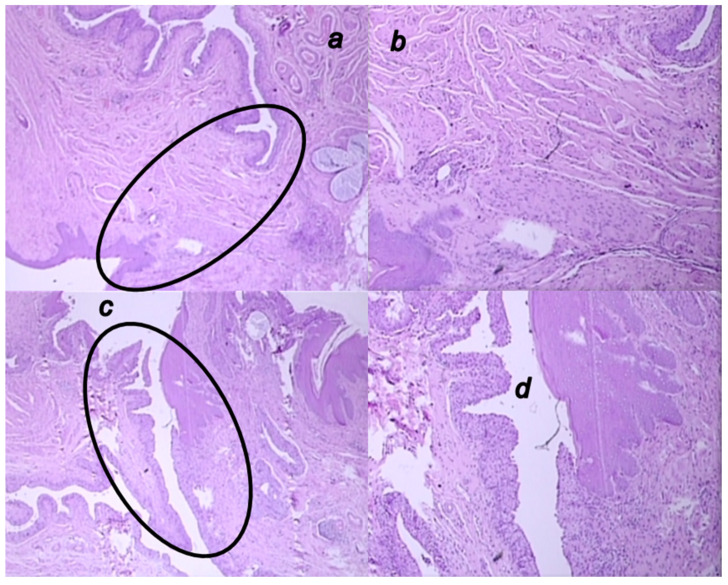
Microscopic presentation of eight-week vesicovaginal fistula, after the six-week therapy of fistulous rats in the BPC 157 treated rats (**a**,**b**) and control rats (**c**,**d**). (**a**,**b**) Visible mature epithelialization, formed epithelium, closed fistula (circle), fully formed collagen fibers and significant neovascularization with the formation of new blood vessels and mature granulation tissue in the BPC 157-rats (HE; ×20 (**a**), ×100 (**b**)). (**c**,**d**). Visible open vesicovaginal fistula code (circle) with moderate inflammation of the fistula’s wall in the controls (HE; ×20 (**c**), ×100 (**d**)).

At the end, in the therapy and fistula time line, this means a tissue defect at the site of fistula with pronounced inflammation (mononuclear and polymorphonuclear) with significant stroma edema in controls, unlike closure of the defect at the fistula site with mild inflammation of the epithelium and stroma in BPC 157 treated rats (Figure 12, Figure 13 and Figure 14).

## 4. Discussion

In principle, beneficial effects noted in the present study support that the BPC 157 therapy for the healing of various both external [5,6,7,8] and internal [9,10] rat fistulas is effective also for the rat vesicovaginal fistulas, continuous urine leaking through vagina, and bladder stones. On the other hand, formation and growth of the stones in the rats with vesicovaginal fistulas provides conclusive evidence of a downhill course, and fistulous rats sharing all predisposing factors in the increasing rate and urinary stasis, from the very early stage until the end of the experiment.

Likewise, the rat vesicovaginal fistula itself as the cause may be important. This may discharge the variety of substances or other surgical procedures leading to urinary calculi in rats and/or mice. There were administered chemicals, acting directly (e.g., melamine) [40] or indirectly (i.e., through metabolites (diethylene glycol leading to calcium oxalate) [41] or through endogenous metabolic product (glycine leading to orotic acid) [42]) as well as the formation of urate calculi following surgical portocaval shunts in rats [43]). Also, in the rat vesciovaginal fistulas, like with the rat rectovaginal fistulas [10], the rat defect size/vaginal size ratio may be close to human disturbance (rectovaginal fistulas are considered complex if they are large (˂2.5 cm) [44]). Together, these rat vesicovaginal fistulas may discharge the vesicovaginal fistulas formation models in other species, i.e., pigs and dogs, providing 14 cm vaginal length, inserted stents to maintain persistent fistulas, 1 cm defect, and no reported stone formation [45,46].

On the other hand, providing the final positive outcome after quite delayed initiation of the therapy, for the BPC 157 beneficial effect, it may be essential that in the fistulous rats BPC 157 administration would instantly reverse the regular noxious course, and this reversal goes toward the complete healing and vesicovaginal fistula closure. Importantly, this is going from the starting point of regular ‘no return’ (i.e., without therapy, two-week vesicovaginal fistulas inevitably result with microlithiasis in the following three weeks (fistula time—week 5) and stone formation in the subsequent weeks). Thus, the therapy that achieved the reversed end point, the vesicovaginal fistula closure and no stone formation, consistently ascertained simultaneous healing of the two tissues and their function maintenance, as it was the case in the previous fistulous studies [5,6,7,8,9,10]. This means in the BPC 157 treated fistulous rats the vaginal and vesical integrity, in fact, recovered. Evidently, this occurred even before the gross, microscopical, and most importantly functional integrity, would be definitively reestablished. In support, BPC 157 shares both vesical integrity and vaginal integrity. Illustratively, in the colovesical fistulas, rats presented gross, microscopical, and functional recovery (lack of the fecaluria) [9]. Rats underwent transabdominal urethrolysis and prolonged vaginal dilatation have attenuated stress urinary incontinence [38]. BPC 157 therapy competes with the cyclophosphamide-induced hemorrhagic cystitis [39]. Likewise, in rat rectovaginal fistulas, BPC 157 healed a large vaginal defect (5 mm (rectovaginal fistulas) vs. 4 mm (vesicovaginal fistula) vs. 2.4 cm vagina length), and abrogated fecal leaking through vagina [10]. Taken together, it is conceivable that already in the first days of the therapy in the rats with vesicovaginal fistulas, BPC 157 eliminated spontaneous urine leaking (upon light rat abdominal pressure) through vagina. This was associated with the increased epithelialization, collagenization, granulation, and neovascularization; decreased inflammation, necrosis, and adhesion formation; as well as the decrease of the defects, vaginal and vesical, and increase of the volume that can be sustained before leaking (which may be four, five times more than in controls), which appeared in the following two weeks of the therapy.

Whatever the pathology background—human fistulas and these rats fistulas have in common a marked fistulas leaking, poor or no healing, and even devastating consequences, and thereby, the significance of the BPC 157 beneficial effects in the fistulas models, the proposed mechanisms from patients vesicovaginal fistulas include vaginal ischemia [14]. Of note, this was not especially investigated in the present study. However, considering the proposed essential role of the ischemia in the falling course of the vesicovaginal fistulas [14], it may be stated that BPC 157 exhibits a particular effect on blood vessels [1,2,3,4], and may rapidly activate collateral pathways to circumvent major vessel occlusion, and reestablish blood flow and compensate otherwise imminent ischemic consequences. There is rapid counteracting effect noted in various severe permanent occlusion of major vessels-induced occlusive syndromes [47,48,49,50,51,52]. Likewise, BPC 157 maintained thrombocytes function (without interference with coagulation) [53,54,55], prevented thrombosis formation, and abrogated already advanced thrombosis that may be associated with vessels lesions [56] or occlusion [47,48,49,50,51,52], arterial [36,50,51], or venous [47,48,49,51,52] as well as arterial and venous [51], peripherally [47,48,49,50,51], or centrally [52]. These vascular effects that may be especially relevant for the fistulas healing [3], were associated with the particular effect on several molecular pathways [18,19,20,21,22,23,24,25,26,27], in particular, having a modulatory effects on NO-system and prostaglandins-system [56,57] and on vasomotor tone and the activation of Src-Caveolin-1-eNOS pathway [21]. Besides, BPC 157 acts as stabilizer of cellular junctions [19], and free radical scavenger [39,58,59], and counteracted free radicals formation and lesions, in particular, in vascular occlusion studies [47,48,49,50,51,60,61,62,63]. Illustratively, the BPC 157 activity as stabilizer of cellular junction (counteracted leaky gut syndrome) goes via increasing tight junction protein ZO-1 expression, and transepithelial resistance [19]. Inhibited were mRNA of inflammatory mediators (iNOS, IL-6, IFNγ, and TNF-α), increased expression of HSP 70 and 90, and antioxidant proteins—such as HO-1, NQO-1, glutathione reductase, glutathione peroxidase 2 and GST-pi [19]. Together, this might be important in providing the reversed course of the vesicovaginal fistulas toward the complete healing. Of note, the additional healing BPC 157’s mechanisms in the vesicovaginal fistulas healing need to be further defined. However, the advanced healing (and collagen) process [5,6,7,8,9,10] fairly reflects the biomechanical improvement [5,6,7,8,9,10], and vice versa. Consequently, the successful instillation of the five times larger volume definitively indicates the rescued fistulas and the healing in BPC 157 rats. Accordingly, the noted attenuated adhesions, stopped urine leaking through vagina in the vesicovaginal fistulas-rats are along with the advanced biomechanical healing commonly noted in the various wounds, fistulas or after surgery, remaining intestine function recovery in BPC 157-studies (for review see, i.e., [3,4]). Likely, the neovascularization, angiogenesis, appear as an essential point for fistulas healing in particular, since pentadecapeptide BPC 157 heals corneal ulcerations in rats, allowing them to regain corneal transparency [64]. The combining point is also the pentadecapeptide BPC 157 given protocol, and its use also in the previous studies [5,6,7,8,9,10] that may fairly mimic human injury, parenterally, or perorally.

Finally, considering the presented and previous findings in fistula research [5,6,7,8,9,10], this study should overwhelm the general point that animal studies per se may be cautious, and fistulas studies; in particular, regarding their results and the relative paucity of the BPC 157 clinical data [1,2,3,4]. On the other hand, BPC 157 was proved to be efficacious in the ulcerative colitis, both in clinical settings [65,66], and in the experimental rats studies, in the ischemic/reperfusion vascular ulcerative colitis studies [60] and other ulcerative colitis models, induced by TNBS [67], cysteamine, surgery [31,68,69], NSAIDs [30,70,71,72,73], or major vessel occlusion [48,49,50,51,52,59], including various species [74], and fistulas as complications (for review see, i.e., [3]). A particular point for revealing and applying this concept in practice (BPC 157’s beneficial effect on various fistulas toward counteraction of the vesicovaginal fistulas in particular) is a very safe profile (LD1 could be not achieved) [17], a point recently confirmed in a large study of the Xu and collaborators [75]. Finally, there are consistently effective used range of BPC 157 (µg-ng) application and used regimens [5,6,7,8,9,10], which may support each other effects, and interestingly, also in the rats with the vesicovaginal fistulas, the same beneficial effect of the application, daily intraperitoneal or peroral in drinking water. Together, these findings (for review see, i.e., [17]) may be suggestive for a physiological role (in situ hybridization and immunostaining BPC 157 in human gastrointestinal mucosa, lung bronchial epithelium, epidermal layer of the skin, and kidney glomeruli) [17]. In this context, the role of the animal model is indispensable, the practical indicative evidence is even more important.

Thus, BPC 157 and resolved vesicovaginal fistula issue—elaborated in this study, along with the other fistulas’ healing [5,6,7,8,9,10]—would certainly indicate BPC 157 as a noteworthy agent for fistula healing. In general, the development of these fistulas models resulted from efforts to improve on agent’s healing spectrum of healing action and stay ahead of the spread of failed healing in fistulas’ course [3]. For the vesicovaginal fistulas, in particular, BPC 157′s easy drug regimen—parenterally or perorally, with suited drug costs, and thereby also improved vesicovaginal fistula surgery—would contribute to the additional resolving research, including clinical studies.

## Data Availability

The data presented in this study are available on request from the corresponding author.
